# The dynamics of ideology drift among U.S. Supreme Court justices: A functional data analysis

**DOI:** 10.1371/journal.pone.0269598

**Published:** 2022-07-08

**Authors:** Xiner Zhou, Hans-Georg Müller

**Affiliations:** Department of Statistics, University of California, Davis, Davis, CA, United States of America; ICREA and Department of Chemical Engineering, Universitat Rovira i Virgili, 43007 Tarragona, Catalonia, SPAIN

## Abstract

We study the U.S. Supreme Court dynamics by analyzing the temporal evolution of the underlying policy positions of the Supreme Court Justices as reflected by their actual voting data, using functional data analysis methods. The proposed fully flexible nonparametric method makes it possible to dissect the time-dynamics of policy positions at the level of individual Justices, as well as providing a comprehensive view of the ideology evolution over the history of Supreme Court since its establishment. In addition to quantifying individual Justice’s policy positions, we uncover average changes over time and also the major patterns of change over time. Additionally, our approach allows for representing highly complex dynamic trajectories by a few principal components which complements other models of analyzing and predicting court behavior.

## Introduction

In view of the dysfunction of the U.S. Congress in recent years, the importance of the Supreme Court may be more critical than ever. With the death of Justice Ginsburg just weeks before the 2020 presidential election and notwithstanding Justice Breyer’s recent decision to retire, the ideological direction of the Supreme Court is likely to change drastically. The appointment of Justice Amy Coney Barrett, the recent confirmation of Justice Ketanji Brown Jackson to fill Justice Stephen Breyer’s vacancy, and the unprecedented leak of a draft opinion that would strike down Roe v. Wade have put the Supreme Court once again in the spotlight and given rise to renewed intense scrutiny.

The study of the ideologies of Supreme Court justices has attracted many scholars [1, 2]. Due to its inherently latent nature, many approaches have been taken to quantify judicial ideologies [3–10]. One of the most popular quantifications is the Martin-Quinn score [10], which introduced a Bayesian item response model to estimate so-called “ideal points”. The literature on the voting behavior of Supreme Court justices [11–13] generally postulates that preferred policy positions of justices are the key explanatory variables of realized voting behavior. These policy positions reflect the underlying latent preferences of justices, with their voting behaviors resulting from these latent factors along with other factors pertaining to the specific cases to be decided and additional extraneous factors. For instance, the “attitudinal model” theorizes that justices vote according to their true attitudes [14]. Based on this postulated relationship between voting behavior and latent ideology, we take here an approach that utilizes the direction (conservative versus liberal) of the observed votes to infer the latent ideologies. Our approach is related to that of [4] using the percentages of liberal votes in a single policy area and that of [10] using a Bayesian item response model.

The proposed approach is based on Functional Data Analysis (FDA) [15–17], a powerful nonparametric statistical methodology that to date has not been much used in the study of law and courts, but has become increasingly popular for the analysis of longitudinal studies or panel data (time-series cross-sectional (TSCS) data) [18–21]. In a nutshell, FDA is a methodology to model the dynamic behavior of an underlying latent stochastic process over a continuum such as time, while the available observations are noisy observations of the process, collected on a discrete and possibly incomplete grid over the domain. Specifically, it is assumed that each justice has a latent policy position that changes continuously over time. We regard this as an instantiation of a latent policy position process, assuming that a justice’s voting behavior is a direct manifestation of this underlying latent process, possibly distorted by some temporaneous influencing factors such as specific features of a case. A similar assumption is also utilized by [10]. The data consist of repeatedly observed votes for each justice and are binary (yes -no) on a dense temporal grid; as we demonstrate, FDA is uniquely suited for the analysis of such data.

One of the benefits of our approach is its ability to compress high-dimensional complex trends into a few variables, the functional principal components, which facilitates further modeling. For instance, judicial behavior might be an important explanatory factor for other political phenomena; for the purpose of predicting Supreme Court votes [22, 23], past judicial behavior can be summarized by functional principal components which can serve as features for machine learning models. Thus, the proposed approach complements existing work related to judicial ideology and voting behavior. Another benefit is that it opens the door to a suite of statistical models analyzing trajectory data in the social sciences.

Using the proposed methodology, we attempt to shed some light on a few questions of interest: Do the policy preferences of U.S. Supreme Court justices change over time? Can patterns be discerned in the policy preference trajectories for justices throughout their tenure and in their individual relative position in the Court? What is the overall dynamics of the Court both in the past and present? And do justices tend to express ideology differently through their voting behavior for different issues, such as civil rights or economic activity?

The paper is organized as follows. In the next section, we briefly describe the Supreme Court Database that forms the starting point for our analysis. This is followed by a very brief introduction to functional principal component analysis and a section on results, where the proposed estimation methods and main results are presented. The paper concludes with a discussion.

## Data

The Supreme Court Database (SCDB) was accessed on January 8th, 2022 [24]. The SCDB includes two releases: SCDB Modern and SCDB Legacy. The most recent SCDB Legacy release is dated October 1st 2021 and contains terms from 1791 to 1945 and contains 172,213 justice-votes records. The most recent SCDB Modern release dated September 30th 2021, contains data from 1946 to 2020 and 122,754 justice-votes records. In total, 294,967 records at the justice-vote level are available between 1791 and 2020.

Since the Supreme Court was established in 1789, 115 justices have served on the Court. As of January 8, 2022, the SCDB (Modern and Legacy combined) contains votes from all justices except for the recently tenured Justice Barrett. Since Thomas Johnson only served for 163 days on the Supreme Court, he was also excluded from the analysis. Consequently, all 113 justices with voting records of more than 163 days are included in the analysis, which thus covers essentially all votes of the justices since the establishment of the Supreme Court.

The justice centered data include an indicator of whether a participating justice cast a liberal or conservative vote along with the date when the vote was cast. These data form the observations on which our analysis is based.

Cases are further labeled according to larger issue areas, which include criminal procedure, civil rights, First Amendment, due process, privacy, attorneys’ or governmental officials’ fees or compensation, unions, economic activity, judicial power, federalism, interstate relations, federal taxation, miscellaneous, and private law. These labels make it possible to conduct a more detailed analysis, as the preferred policy positions of justices may manifest themselves differently in different case categories.

## Methods

### Basic approach

The main methodology for our analysis is based on Functional Data Analysis (FDA) [16, 25]. In FDA, curve data are viewed as sample paths of a continuous time stochastic process over some continuum, usually a common time interval. In the present context, a sample path *X*(*t*) corresponds to a justice’s latent policy preference process, or ideology process, as it evolves over time within a fixed common time interval T.

Since we are interested in the evolution of justices’ ideology, in a preprocessing step we take the origin of time *t* = 0 for each justice as the time at which a justice was appointed. Accordingly, the trajectories for all justices are defined on the same time interval T and the ideology process is viewed as evolving over “time since tenure”. The observed end point of this continuum, however, varies from justice to justice, because justices serve on the Court for different lengths of time; the end of their tenure is random and may be due to resignation or death. The length of service on the Court for the 106 non-incumbent justices ranged from William O. Douglas’s 36 years and 211 days to the 163-day tenure of Thomas Johnson. As of January 9, 2022, the length of service for the nine incumbent justices ranges from Thomas’ 30 years and 78 days to Barrett’s 1 year and 74 days. The median, first quantile and third quantile of length of service are 24, 16, and 30 years, respectively. Due to the rich information available through SCDB, we were able to select the relatively long time interval T=[0,35] years since appointment as the common time interval for the analysis.

It is then of interest to study how the justice’s policy preferences *X*(*t*) change throughout the tenure of the justice on the Court on the time interval T=[0,35] years since appointment. One may always visualize the results in calendar time if desired.

A challenge is that only William Douglas had a tenure period of more than 35 years, and 8 other justices had tenures that are just shy of 35 years. For these justices their voting behavior is observable over the entire time interval T=[0,35] and thus they have completely observed functional data. For all the other justices with less than 35 years of tenure, the functional data are partially observed as data towards the right end of the time interval are not available for these justices, and the shorter their tenure period is the more data are missing.

The Supreme Court Database features data of the form {(*t*_*ij*_, *Y*_*ij*_): *i* = 1…113, *j* = 1…, *m*_*i*_}, where there are *n* = 113 justices and the *i*-th justice has *m*_*i*_ recorded votes. Here *t*_*ij*_ refers to the time when the *j*-th decision is recorded for the *i*-th justice, measured in terms of days since appointment of the justice. The *Y*_*ij*_ represent the *i*-th justice’s votes at decision time *t*_*ij*_, with *Y*_*ij*_ = 1 if the justice casts a conservative vote and *Y*_*ij*_ = 0 if the justice casts a liberal vote. To address the issue of unequal tenure periods and thus partially observed functional data, we use a statistical model that connects the latent policy position process to the actually observed data for each justice.

The observed votes at day *t*_*ij*_ for the *i*–th justice are assumed to follow a Bernoulli distribution with probability

$$\begin{array}{c} p_{i}\left(t_{ij}\right)=P\left(Y_{ij}=1|T=t_{ij}\right), \end{array}$$ (1)

where the binary observed response at *t*_*ij*_ is the result of the justice’s preferred policy position and additional noise stems from the nature of particular cases or other exogenous or subjective variables that are unknown. The preferred policy position can be considered a latent trait that is expected to be stable in the short-term but may change over time smoothly. This is reflected by our assumption that the functions *p*_*i*_(*t*) are continuously differentiable.

The link between *p*_*i*_(*t*) and the desired ideology process *X*_*i*_(*t*) can be modeled by hypothesizing that ideology of justices is the key explanatory variable for realized voting behavior while other factors pertaining to the specific cases to be decided and additional extraneous factors may play an additional role. This hypothesis is adopted by many scholars [4, 10–14]. Based on this relationship between voting behavior and latent ideology, we build the following model to infer the latent ideology process *X*_*i*_(*t*) from the observable decisions *p*_*i*_(*t*_*ij*_).

We link the probability of an observation of a “1” outcome and the latent (Gaussian) process via a logit transformation as follows,

$$\begin{array}{c} \text{logit}\left(p_{i}\left(t_{ij}\right)\right)=\text{log}\frac{p_{i}\left(t_{ij}\right)}{1-p_{i}\left(t_{ij}\right)}=X_{i}\left(t_{ij}\right)+\epsilon_{ij},\;t_{ij}\in T,\; \end{array}$$ (2)

where errors *ϵ*_*ij*_ denote local aberrations from the smooth underlying processes *X*_*i*_(*t*). The logistic transform has the effect to transform the functions *p*_*i*_(*t*) which are restricted by 0 < *p*_*i*_(*t*) < 1 to *X*_*i*_(*t*), which are unrestricted real-valued functions as required by FDA methodology. This approach does not rely on any assumption about the time-varying or constant nature of the ideology of Supreme Court justices over the time domain. Our methodology embodies the principle of “letting the data speak for themselves” by imposing only minimal assumptions. Thus it is ideally suited to provide empirical evidence for the debate whether judicial preferences are constant or changing over time [26–31].

To summarize, the estimation of the latent policy position processes $\left\{X_{i}\left(t\right):\;i=1\ldots n,t\in T\right\}$ follows two steps. The first step is to transform the binary observations {(*t*_*ij*_, *Y*_*i*_(*t*_*ij*_)): *i* = 1, … *n*, *j* = 1, …, *m*_*i*_} into functional data $\left\{\text{logit}\left({\hat{p}}_{i}\left(t\right)\right):\;i=1\ldots n\right\}$. In a second step, we apply Functional Principal Component Analysis (FPCA) to the functional data $\left\{\text{logit}\left({\hat{p}}_{i}\left(t\right)\right):\;i=1\ldots n\right\}$, aiming to estimate the underlying latent policy processes $\left\{X_{i}\left(t\right):\;i=1\ldots n,t\in T\right\}$ for each justice.

### Converting binary observations to functional data

To convert the binary observations {(*t*_*ij*_, *Y*_*i*_(*t*_*ij*_): *i* = 1, …, *n*, *j* = 1, …, *m*_*i*_} into functional data, the starting point is to obtain smooth probability functions in time *t*, over the time period for which one has data for an individual justice. To this end, we observe that *p*_*i*_(*t*) = *P*(*Y*_*i*_(*t*) = 1) = *E*[*I*{*Y*_*i*_(*T*) = 1}|*T* = *t*] and that this conditional expectation can be viewed as a regression function over the time domain, an estimate of which can then be obtained by scatterplot smoothing. For this smoothing step, we adopt Local Linear Smoothing (LLS) [32, 33] to obtain a continuous estimated probability function ${\hat{p}}_{i}\left(t\right)$ for each justice, where a smoothing bandwidth of *h* = 365 days was used to borrow information from neighboring cases within one year. This choice of the smoothing bandwidth avoids situations where there are too few cases at the Court during a shorter period of time, which would lead to highly variable estimates. Thus with this choice one obtains practically interpretable results. We also found that the results were not overly sensitive to the choice of the smoothing bandwidth.

To facilitate the subsequent application of the logit transform, which requires ${\hat{p}}_{i}\left(t\right)$ to be strictly larger than 0 and smaller than 1, we introduce a small threshold *ρ* > 0 such that the function estimates ${\hat{p}}_{i}\left(t\right)$ are always in the interval [*ρ*, 1 − *ρ*]. This can be achieved by setting values that fall outside this interval to equal the closer one of these boundary points, where *ρ* = 0.001 was chosen as this value was found to be adequate to constrain the values of ${\hat{p}}_{i}\left(t\right)$ away from 0 or 1 but only by a negligible amount. We then obtained the set of measurements of the underlying latent trajectories with additive noise as

$$\begin{array}{c} Z_{ij}=\text{logit}\left({\hat{p}}_{i}\left(t_{ij}\right)\right):\;i=1,\ldots ,n,\;j=1,\ldots ,m_{i}. \end{array}$$ (3)

These are considered measurements of the underlying unknown trajectories *X*_*i*_(*t*) at time points *t*_*ij*_ that may carry noise due to aberrations from the smooth underlying trajectories when the vote was taken. While we assume here that the combination of a smoothing step followed by a logit transformation leads to potentially still noisy measurements of an underlying smooth process, which is vindicated by the practical success of this approach, nonparametric alternatives where the link function is not specified could also be considered [34].

After these pre-processing steps, the resulting data are {(*t*_*ij*_, *Z*_*ij*_):*i* = 1, …, *n*, *j* = 1, …, *m*_*i*_}. These are assumed to be related to the underlying latent policy preference processes through

$$\begin{array}{c} Z_{ij}=X_{i}\left(t_{ij}\right)+\epsilon_{ij},\;i=1,\ldots ,n,\;j=1,\ldots ,m_{i}. \end{array}$$ (4)

Here the errors *ϵ*_*ij*_ are assumed to be mean zero finite variance (Gaussian) random variables with $EZ_{ij}^{2}=\sigma_{\epsilon }^{2}$ that reflect estimation errors and noisy oscillations that are not part of the smooth latent trajectories *X*_*i*_(*t*).

### Functional principal component analysis (FPCA)

Our goal in this step is to estimate the latent policy position processes {*X*_*i*_(*t*): *i* = 1…113} on a domain T=[0,35] years since tenure by applying Functional Principal Component Analysis (FPCA) to the data {(*t*_*ij*_, *Z*_*ij*_): *i* = 1, …, *n*, *j* = 1, …, *m*_*i*_}, which are noisy realizations of the underlying ideology process.

FPCA is based on the eigendecomposition of the Hilbert-Schmidt linear operator with covariance kernel *C*(*s*, *t*) = cov(*X*(*s*), *X*(*t*)), leading to the decomposition *C*(*s*, *t*) = ∑_*j*≥1_ λ_*j*_ *φ*_*j*_(*s*)*ϕ*_*j*_(*t*) with eigenvalues λ_*j*_ > 0, λ_1_ > λ_2_ > … and a sequence of orthonormal eigenfunctions *ϕ*_*j*_, *j* ≥ 1. Under mild assumptions, this entails the Karhunen-Loève representation of trajectories *X*_*i*_(*t*) [25] given by

$$\begin{array}{c} X_{i}\left(t\right)=\mu \left(t\right)+\sum_{j=1}^{\infty }\xi_{ij}\phi_{j}\left(t\right), \end{array}$$ (5)

where *ξ*_*i*1_, *ξ*_*i*2_, … are mean zero uncorrelated functional principal components (FPCs), often referred to as FPC scores, with explicit representation as integrals (inner products) *ξ*_*ij*_ = ∫(*X*(*t*) − *μ*(*t*))*ϕ*_*j*_(*t*)*dt* and variances var(*ξ*_*ij*_) = λ_*j*_, *j* = 1, 2, ….

In practice, the expansion is approximated by only including the first *K* components in the sum on the right hand side of Eq (5), where *K* is typically chosen to achieve a large fraction of the variation explained, most commonly FVE = .95 or FVE = .99, where here we choose the latter. The mean function *μ*(*t*) and the eigenfunctions, eigenvalues and principal components can be estimated with the PACE algorithm [16, 18, 35] which is available in the R package fdapace [36].

It is important to note that for the PACE implementation of FPCA one does not require data for individual justices to be available on the entire domain [0, *T*], rather it is required that the pairs of times where repeated measurements are obtained for the same justice when plotted against each other, for all justices combined, will densely fill the square [0, *T*]^2^, which can be ascertained by a domain plot [18]. If this condition is satisfied, then assembling the voting data for justices with both longer and shorter tenures will still lead to consistent estimates of the eigencomponents of the FPCA over the entire domain [0, *T*], as long as one has data from a sufficient number of justices whose tenure exceeds [0, *T*].

Once the eigencomponents on the entire interval [0, *T*] have been obtained, for *K* selected components, the estimated FPCs for the *i*–th justice and *k*–th component (*i* = 1, …, *n*; *k* = 1, …, *K*) can be obtained as best linear predictors [18],

$$\begin{array}{c} {\hat{\xi }}_{ik}=\hat{E}[\xi_{ik}|{\tilde{Z}}_{i}]={\hat{\lambda }}_{k}{\hat{\phi }}_{ik}^{T}{\hat{\Sigma }}_{Z_{i}}^{-1}({\tilde{Z}}_{i}-{\tilde{\mu }}_{i}), \end{array}$$ (6)

where ${\tilde{Z}}_{i}$ is a vector containing the measurements of the underlying latent trajectories with additive noise *Z*_*ij*_ (see Eq (4)) for the *i*-th justice evaluated at the justice-specific times *t*_*ij*_, with ${\tilde{\mu }}_{i}$ denoting a vector of the overall means, also evaluated at the justice-specific times *t*_*ij*_. Furthermore, $\Sigma_{Z_{i}}=\text{cov}\left({\tilde{Z}}_{i}\right)=\text{cov}\left({\tilde{X}}_{i}\right)+\sigma_{\epsilon }^{2}I_{m_{i}}$ is the covariance matrix of the observed data ${\tilde{Z}}_{i}$. To implement the PACE algorithm we used the R package fdapace [36].

We then insert the estimated FPCs obtained from Eq (6) into the representation Eq (7), which yields the estimated trajectory ${\hat{X}}_{i}\left(t\right)$ on [0, *T*] for the *i*-th justice.

The guiding principle is to gain strength by pooling the data from all justices when representing the trajectory of individual justices. When choosing *K* components, substituting these estimates leads to the representation

$$\begin{array}{c} {\hat{X}}_{i}\left(t\right)≔\hat{\mu }\left(t\right)+\sum_{j=1}^{K}{\hat{\xi }}_{ij}{\hat{\phi }}_{j}\left(t\right), \end{array}$$ (7)

where ${\hat{X}}_{i}\left(t\right)$ is the estimated ideology process for the *i*-th justice from time of appointment to up to 35 years of tenure period.

This representation can be easily transformed to the probability scale (between 0 and 1) for the likelihood of conservative votes, aiding a more transparent interpretation, by applying the expit transformation which is the inverse of logit, i.e.,

$$\begin{array}{c} {\tilde{p}}_{i}\left(t\right)=\text{expit}\left({\hat{X}}_{i}\left(t\right)\right)=\frac{\text{exp}\left({\hat{X}}_{i}\left(t\right)\right)}{1+\text{exp}\left({\hat{X}}_{i}\left(t\right)\right)},\;t\in \left[0,T\right]. \end{array}$$ (8)

The estimator given in Eq (7) is based on the best linear unbiased prediction principle and there is additional justification for these predictors if the (transformed) trajectories *X*_*i*_ are Gaussian processes. The predicted trajectories are unbiased if *K* is sufficiently large [18].

#### Interpretation of the mean and eigencomponents

The estimated mean function $\hat{\mu }\left(t\right)$ represents the average ideology process on the domain [0, 35] years for the 113 Supreme Court justices. The estimated eigenfunctions ${\hat{\phi }}_{j}\left(t\right),j=1\ldots K$ represent the leading patterns of variation in the ideology processes extracted from the observed voting behavior and reflect the major ways of ideology change over time for the justices. A flat eigenfunction indicates no change over time in the direction of this eigenfunction. The estimated FPCs ${\hat{\xi }}_{ij}$ are justice-specific in contrast to mean and eigenfunctions, which reflect the entire population of justices. They indicate in which way the ideology trajectory of the *i*-th justice moves along over time in the direction represented by the *j*th estimated eigenfunction ${\hat{\phi }}_{j}\left(t\right)$, with large positive values reflecting a stronger alignment in the corresponding eigendirection and a negative value reflecting alignment in the opposite direction.

#### Dimension reduction

The representation in Eq (7) establishes a one-to-one correspondence between the latent processes *X*_*i*_(*t*) and *K*-dimensional random vectors that consist of the FPCs *ξ*_*i*1_, …, *ξ*_*iK*_. It is through this correspondence that one achieves dimension reduction for the original highly complex trajectory data to a representation by a *K*-dimensional random vector, where in many cases choosing a dimension *K* between 2 and 4 provides a reasonably good low-dimensional approximation. The resulting *K*-vector (*ξ*_*i*1_, …, *ξ*_*iK*_) then represents the trajectory for the *i*-th justice and can then be used for other statistical analysis or machine learning models.

#### Prediction of future ideology process

If the tenure period of a justice is [0, *S*], *S* < 35, then the estimator ${\hat{X}}_{i}\left(t\right)$ presented in Eq (7) on [*S*, 35] is the predicted ideology trajectory for this justice, where the end point *S* corresponds to current time, and all times *t* > *S* are in the future relative to the tenure period of the justice for whom the future ideology trajectory is to be predicted. The prediction relies on pooling information from that specific justice as well as others. The principle of the prediction is to take the data from the justices that are observed on the entire time domain [0, 35] or at least a longer domain and to infer from those data the future voting behavior of a justice for whom data are only observed on a subset [0, *S*] of the total domain [0, 35]. This device has been recently also used for the prediction of COVID-19 case trajectories [37].

The PACE principle [18] giving rise to Eqs (6) and (7) is to pool the data to gain insights into the general modes of variation (which are determined by the eigenfunctions and show the main directions of variation). Eq (7) gives the inferred ideology process over the complete 35 years after tenure. For those justices whose voting data are only available on [0, *S*], *S* < 35, it can also be used to infer their future likely ideology processes, which leads to predictions of potential (and never observable for those with short tenure) trajectories.

## Results

### Time evolution of ideology of justices

**Fig 1:**
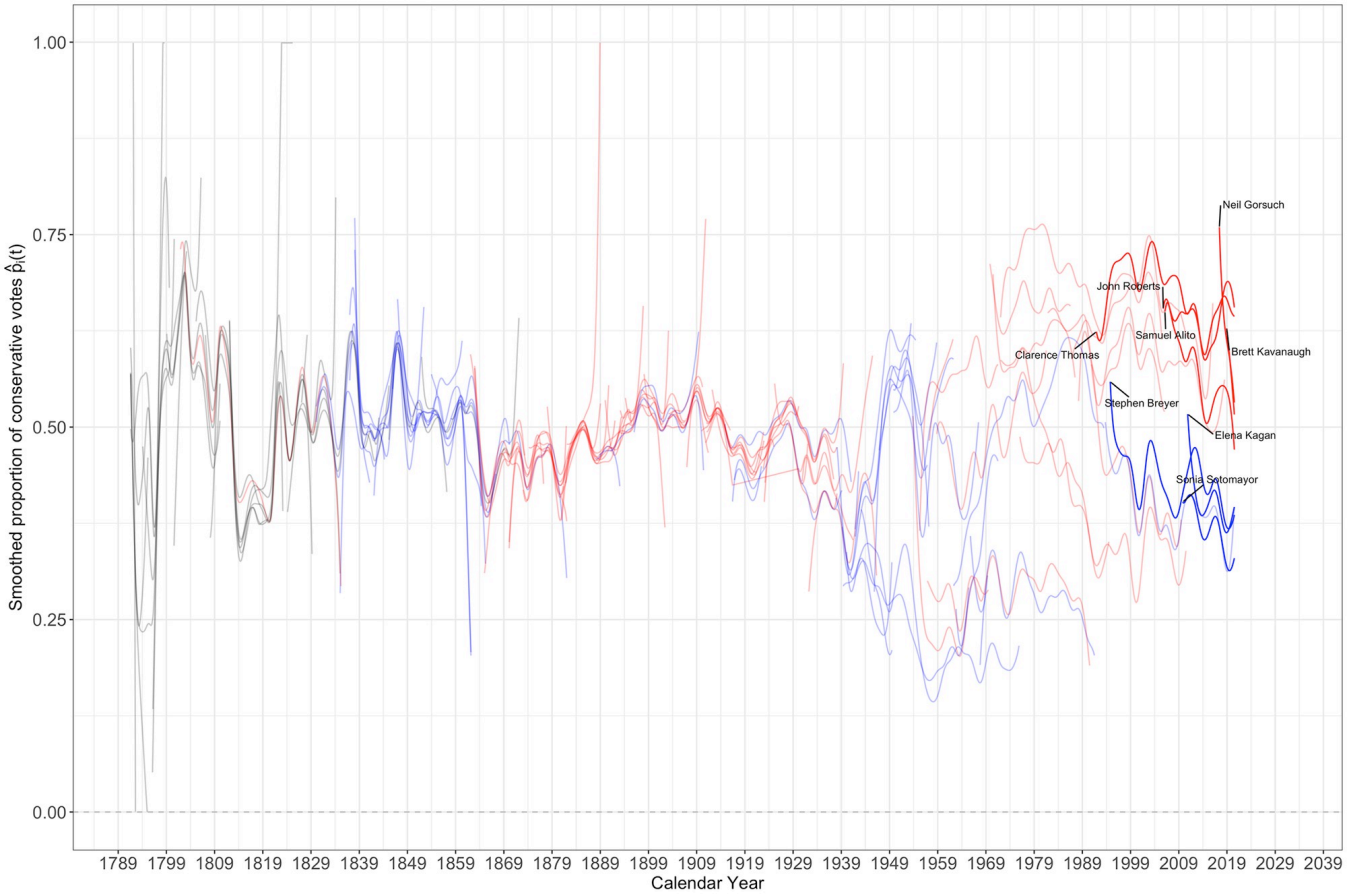
Pre-smoothed curves ${\hat{p}}_{i}\left(t\right),\;i=1,\ldots ,113$ against calendar year. The curves ${\hat{p}}_{i}\left(t\right)$ represent the smoothed proportion of conservative votes as a function of calendar year *t* for the *i*-th justice and thus the ideology of the 113 justices in the sample. The curves are color coded by the nominating president’s party affiliation: red stands for a justice nominated by a Republican, blue for a justice nominated by a Democratic president and gray for a justice nominated by a third party president. The currently active justices are highlighted.

**Fig 2:**
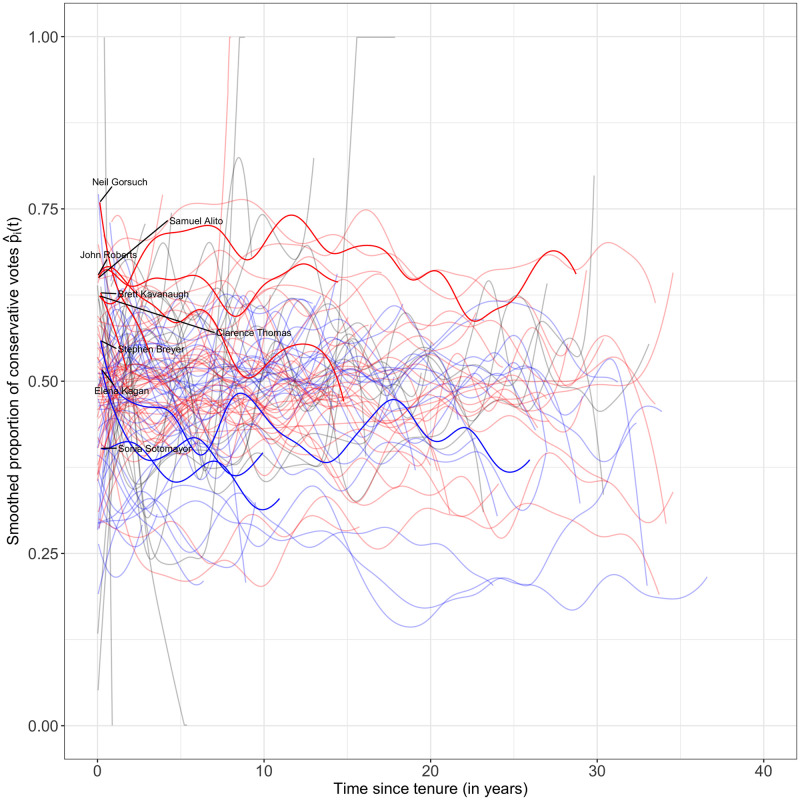
Pre-smoothed curves ${\hat{p}}_{i}\left(t\right),\;i=1,\ldots ,113$ against year since appointment of the justices. Here the curves ${\hat{p}}_{i}\left(t\right)$ represent the smoothed proportion of conservative votes as a function of time *t* after appointment for the *i*-th justice and thus the ideology of the 113 justices in the sample over their tenure period. The curves are color coded by the nominating president’s party affiliation: red stands for a justice nominated by a Republican, blue for a justice nominated by a Democratic president and gray for a justice nominated by a third party president. The currently active justices are highlighted.

After the pre-processing smoothing step, we have a sample of 113 smooth curves $\left\{{\hat{p}}_{i}\left(t\right):\;i=1\ldots 113\right\}$, each representing the observed proportion of conservative votes as a function of time for one justice. These curves are displayed in Fig 1 according to calendar year and in Fig 2 according to year since appointment. The overall picture suggests that, prior to the 1940s, justices tended to vote similarly as their observed voting behaviors were closely aligned; after the 1940s, the discrepancies in terms of voting behavior between justices increased substantially and more recently a clear divide between Democratic and Republican appointed justices began to emerge.

### Ideology dynamics through FPCA

#### The average ideology process and main patterns of change

**Fig 3:**
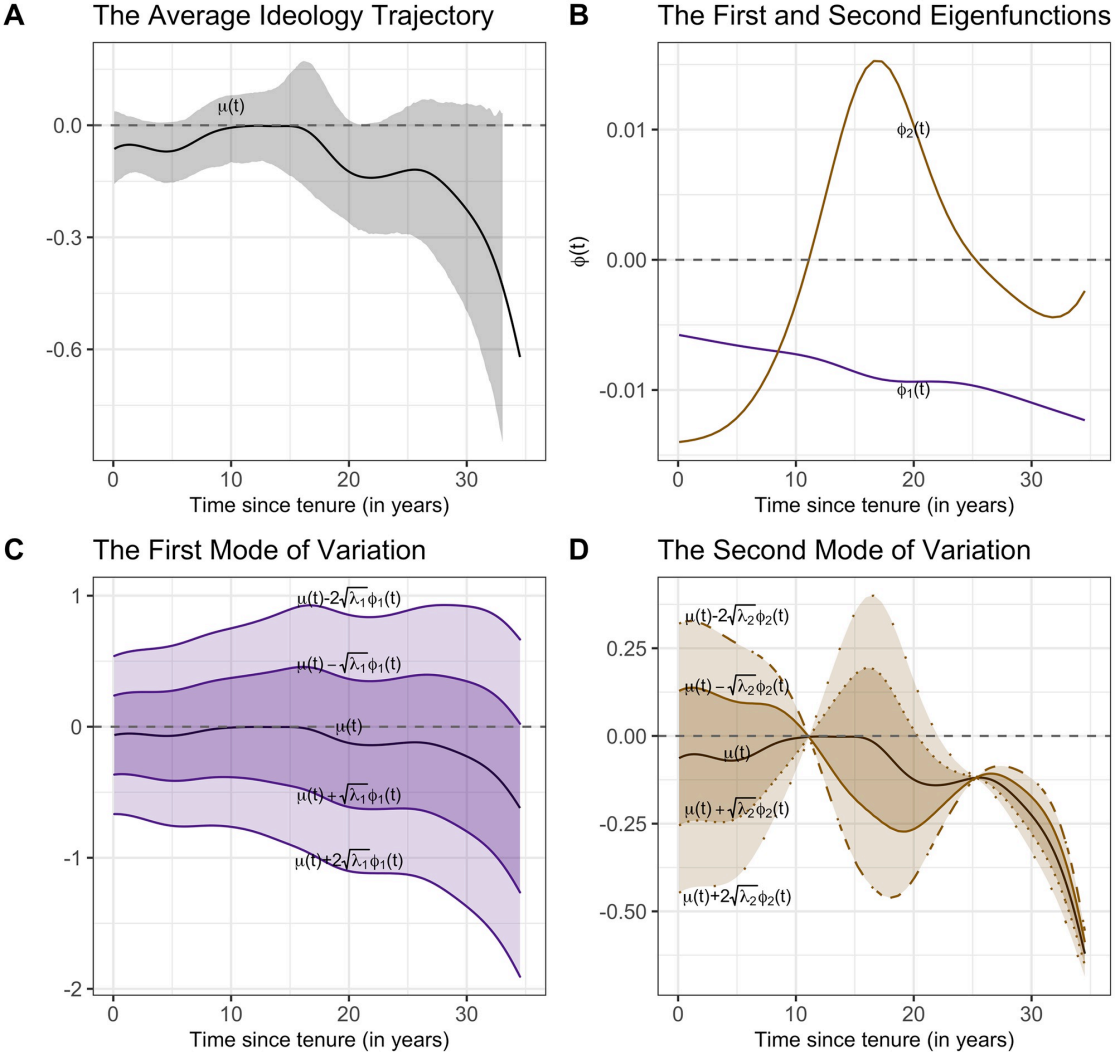
Average ideology trajectory and first two eigenfunctions. A: the estimated average ideology trajectory, with shaded region corresponding to 95% pointwise bootstrap confidence intervals for the actual mean trajectory; B: the first and second eigenfunctions *ϕ*_1_(*t*) and *ϕ*_2_(*t*); C: the first mode of variation $\mu \left(t\right)\pm k\sqrt{\lambda_{1}}\phi_{1}\left(t\right)$ for *k* = 0, 1, 2 where $\sqrt{\lambda_{1}}$ is the standard deviation of the FPCs corresponding to the first eigenfunction; D: the second mode of variation $\mu \left(t\right)\pm k\sqrt{\lambda_{2}}\phi_{2}\left(t\right)$ for *k* = 0, 1, 2 where $\sqrt{\lambda_{2}}$ is the standard deviation of the FPCs corresponding to the second eigenfunction.

Fig 3A shows the mean ideology trajectory across all justices, in the logit scale, so that a probability of 0.5 for a conservative vote corresponds to 0. This suggests that, on average, justices start as centrists for the first 15 years, but then are subject to a slight tendency to a more liberal ideology. This supports the argument that the Supreme Court justices’ ideology does change over time.

The major archetypes of dynamics are extracted by FPCA and presented by the first two eigenfunctions depicted in Fig 3B. The first two eigenfunctions *ϕ*_1_, *ϕ*_2_ explain 97.3% of the total variation, with *ϕ*_1_ accounting for 91% and *ϕ*_2_ accounting for 6.3%. Analogous to multivariate principal component analysis, FPCA projects high-dimensional curve data into a low dimensional space, with each dimension representing a major pattern of change, which in their entirety represent the dynamics of the trajectories.

The first eigenfunction *ϕ*_1_ in Fig 3B represents the first such dimension, and when multiplied with positive factors indicates consistently more liberal positions than the average. The large fraction of variance explained by this first eigencomponent shows that by far the largest source of variation is indeed a basic conservative or liberal policy preference, along with a slight tendency that this preference becomes more expressed as the tenure of a justice wears on.

The second eigenfunction *ϕ*_2_ represents the second dimension, that is, a time-dynamic trend toward the opposite side of policy preference from the starting point after 10 years followed by a moderate swing back toward the original position after 20 years. This component thus reflects a moderate swing dynamics pattern. The second eigenfunction mainly captures how policy preferences swing back and forth to some extend over time of tenure, and this is in contrast to the first eigenfunction, which captures the basic levels of liberalism or conservatism with a deepening trend.

To see how the average trajectory, the first and second eigenfunctions interact to lead to the manifest dynamics, it is helpful to visualize the modes of variation in Fig 3C and 3D. Fig 3C shows the first mode of variation, $\mu \left(t\right)\pm \sqrt{\lambda_{1}}\phi_{1}\left(t\right)$, which is comprised of hypothetical trajectories that lie one standard deviation away from the average trajectory in the dimension represented by the first eigenfunction; and $\mu \left(t\right)\pm 2\sqrt{\lambda_{1}}\phi_{1}\left(t\right)$, lying two standard deviations away from the average trajectory. Clearly, without any additional dynamic provided by the second dimension, trajectories would mostly differ in the overall levels of liberalism or conservatism throughout, where these levels are measured in terms of differences from the average level and intensify slightly over time as tenure progresses. Fig 3D shows the second mode of variation, where the curves $\mu \left(t\right)\pm \sqrt{\lambda_{2}}\phi_{2}\left(t\right)$ depict hypothetical trajectories one standard deviation away from the average trajectory in the dimension represented by the second eigenfunction, while $\mu \left(t\right)\pm 2\sqrt{\lambda_{2}}\phi_{2}\left(t\right)$ depict hypothetical trajectories situated two standard deviations away. The effect of this second dimension is trending toward the opposite side of policy preference from the starting point after 10 years followed by a moderate swing back toward the original position after 20 years.

A unique feature of the FDA approach and especially FPCA is to decompose the observed trajectories into distinct modes of variation and thus gain insights into the main drivers of the observed trends. The orthogonal eigenfunctions thus serve to elucidate different aspects of the observed ideology dynamics. The principle of FPCA thus is to represent complex patterns of change by a few mutually orthogonal patterns. This approach is completely data-driven and one is not limited to any pre-conceived functional forms. While the average trajectory reflects trends that apply to all justices equally, the first two FPCs represent the individual variation across justices, where the FPCs can be understood as random effects in addition to the fixed effect represented by the average trajectory.

#### Justice-specific functional principal components

**Fig 4:**
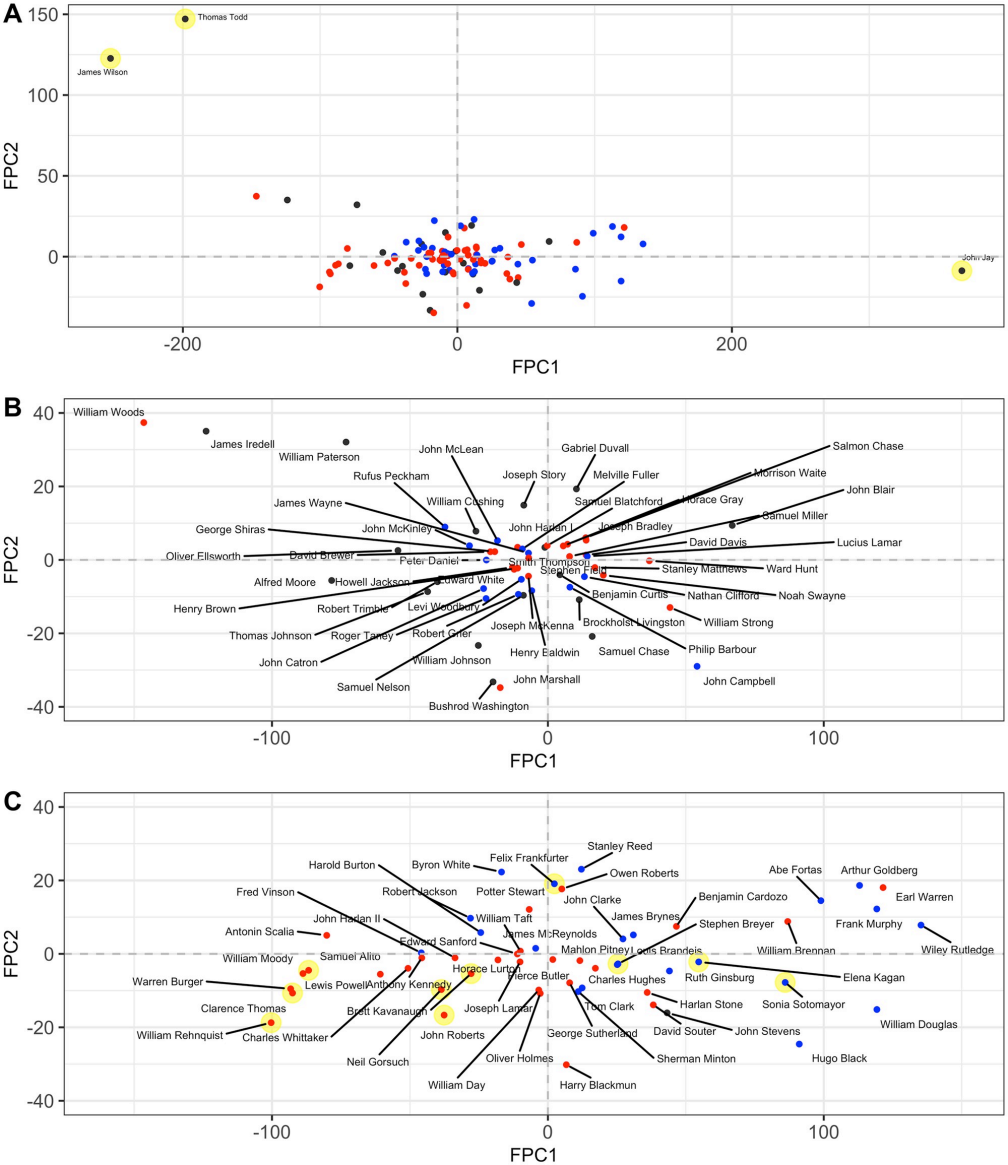
First and second Functional Principal Components (FPCs). Estimated FPCs were obtained as per Eq (6). They visualize the FPC-space representation of ideology trajectories, where coordinates represent the amount of deviation from the average trajectory in the direction of the first and second eigenfunctions. Color coded by the nominating president’s party (blue for Democratic, red for Republican). The justices that are mentioned specifically are highlighted in yellow. (A) exhibits all 113 justices, with 3 obvious outliers: Thomas Todd, James Wilson, and John Jay. (B) exhibits all non-outlier justices before 1900 with annotated names. (C) exhibits all non-outlier justices after 1900 with annotated names.

For most justices the two patterns of variation reflected by the modes of variation are both present, where the first component explains by far most of the variation and thus dominates, while the dynamic pattern reflectedby the second component is less noticeable. The scatterplot of the FPCs in Fig 4 reveals which pattern a justice largely follows.

Different regions in the FPC space represent different ideology dynamics. FPCs of justices with consistently more liberal disposition are located in the right half-plane, and those with consistently more conservative disposition are located in the left half-plane. Justices with a shift toward a more conservative ideology over the course of their tenure in addition to the average trend are located on the upper half-plane, and those with a shift toward a more liberal ideology are located on the lower half-plane.

Clearly interpretable patterns emerge for justices whose FPCs are situated near the main axes. Trajectories of justices whose FPCs are situated close to the *x*-axis generally follow the pattern depicted in the first mode of variation as shown in Fig 3C. Trajectories of justices whose FPCs are situated close to the *y*-axis generally follow the pattern depicted for the second mode of variation in Fig 3D. For example, Felix Frankfurter started as more liberal but had a drastic shift toward a conservative ideology after 10 years. This pattern is confirmed in Fig 8.

**Fig 5:**
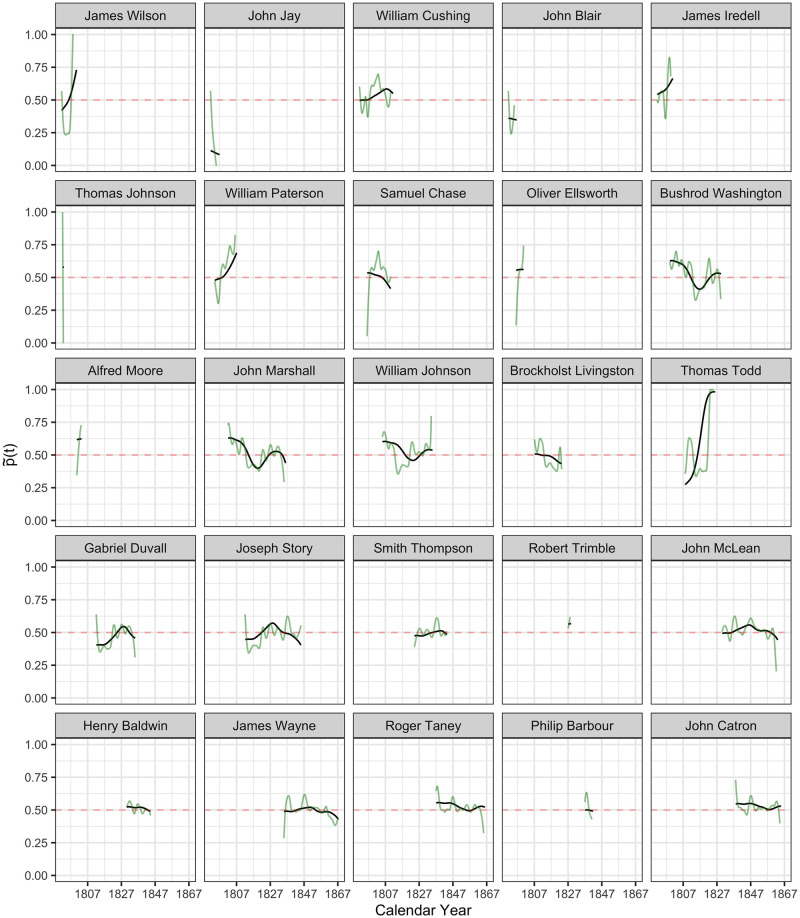
The inferred latent ideology processes ${\tilde{p}}_{i}\left(t\right)$ in probability scale, as per Eqs (7) and (8), from James Wilson to John Catron. Latent ideology processes ${\tilde{p}}_{i}\left(t\right)$ are shown in black and the observed (smoothed) processes ${\hat{p}}_{i}\left(t\right)$ in green, where time *t* is calendar time.

The justices with FPCs near the diagonal or off-diagonal regions exhibit a mix of dynamics that adds to the average trajectory, and show dynamic patterns related to both first and second eigenfunctions. For example, William Rehnquist has very negative FPC1 and FPC2, which means his trajectory is a mix of “consistently more conservative than the average” and “a shift toward liberal direction after 10 years since appointment”. This pattern can be confirmed in Fig 8. An interesting outlier is Thomas Todd who has a very negative FPC1 but a very positive FPC2, which means his trajectory would be a mix of “consistently more conservative than the average” and “a shift toward conservative direction after 10 years since tenure”. This pattern is confirmed in Fig 5.

For simplicity, in the following we will label justices appointed by a Republican (Democrat) president simply as Republican (Democrat). The separation between Republicans and Democrats is clearly visible, where Republicans are clustered on the left half-plane, and Democrats on the right half-plane. This is as expected. We ascertained these effects by simple linear regression models, by regressing *ξ*_1_ and *ξ*_2_ on party affiliation of the appointing president (Republican or Democrat), and calendar year at tenure. The coefficients and associated significance from the two models are reported in Table 1. From the effects on *ξ*_1_, Republicans are significantly more associated with the pattern “consistently more conservative position than the average”, and Democrats with the opposite pattern, again as expected.

**Table 1:** Results from linear regression models.

	FPC1 *ξ*_1_	FPC2 *ξ*_2_
Republican	-35.597***	-2.726
Year after appointment	0.080	0.007

*Note*:

*p<0.1;

**p<0.05;

***p<0.01

Linear regression models are used to assess which factors are associated with observed patterns, by regressing FPC1 *ξ*_1_ and FPC2 *ξ*_2_ on party affiliation (Republican or Democrat), justice’s age at appointment, and calendar year at appointment.

#### Reconstruction of latent policy position processes

**Fig 6:**
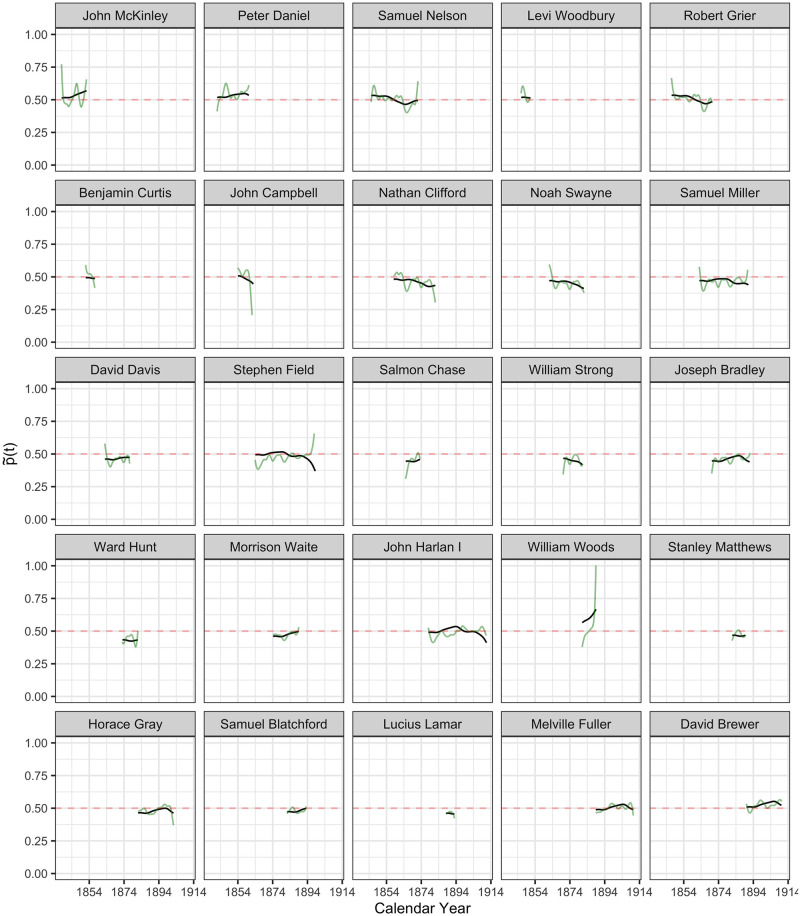
The inferred latent ideology processes ${\tilde{p}}_{i}\left(t\right)$ in probability scale, as per Eqs (7) and (8), from John McKinley to David Brewer. Latent ideology processes ${\tilde{p}}_{i}\left(t\right)$ are shown in black and the observed (smoothed) processes ${\hat{p}}_{i}\left(t\right)$ in green, where time *t* is calendar time.

**Fig 7:**
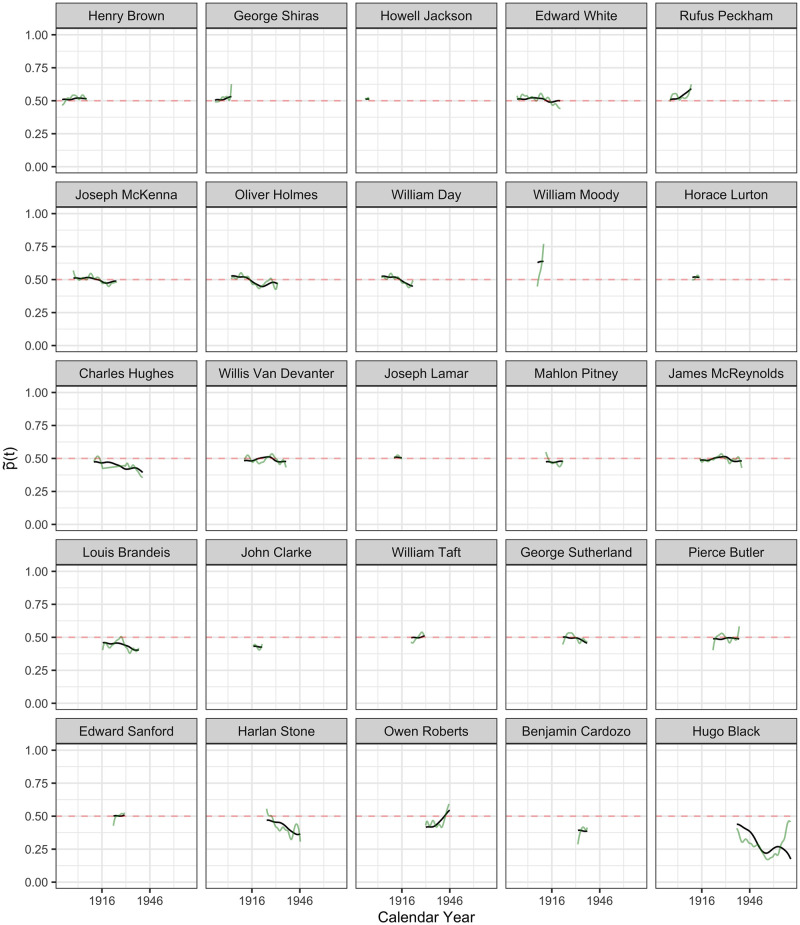
The inferred latent ideology processes ${\tilde{p}}_{i}\left(t\right)$ in probability scale, as per Eqs (7) and (8), from Henry Brown to Hugo Black. Latent ideology processes ${\tilde{p}}_{i}\left(t\right)$ are shown in black and the observed (smoothed) processes ${\hat{p}}_{i}\left(t\right)$ in green, where time *t* is calendar time.

**Fig 8:**
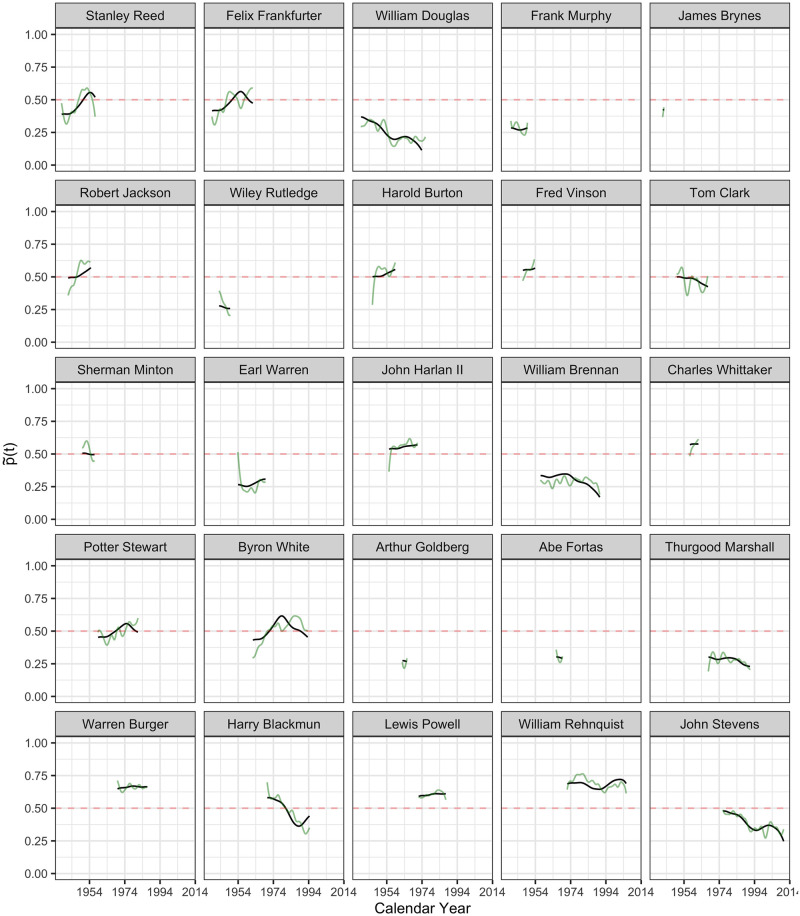
The inferred latent ideology processes ${\tilde{p}}_{i}\left(t\right)$ in probability scale, as per Eqs (7) and (8), from Stanley Reed to John Stevens. Latent ideology processes ${\tilde{p}}_{i}\left(t\right)$ are shown in black and the observed (smoothed) processes ${\hat{p}}_{i}\left(t\right)$ in green, where time *t* is calendar time.

**Fig 9:**
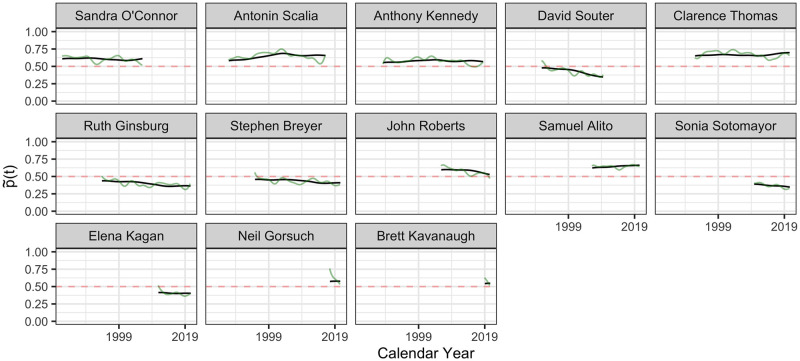
The inferred latent ideology processes ${\tilde{p}}_{i}\left(t\right)$ in probability scale, as per Eqs (7) and (8), from from Sandra O’Connor to Brett Kavanaugh. Latent ideology processes ${\tilde{p}}_{i}\left(t\right)$ are shown in black and the observed (smoothed) processes ${\hat{p}}_{i}\left(t\right)$ in green, where time *t* is calendar time.

The inferred latent ideology processes $\left\{{\hat{X}}_{i}\left(t\right):i=1\ldots 113\right\}$ for the 113 justices, as per Eq (7), shown as functions $\tilde{p}\left(t\right)$ in the probability scale, as per Eq (8), are visualized in Figs 5–9 in calendar time.

#### The current Roberts court

The ideology makeup of the Roberts Court is of obvious current interest. Before Ginsburg passed away, the ranking of the policy preferences of the justices on the current and recent court from most conservative to most liberal was: Thomas, Alito, Gorsuch, Kavanaugh, Roberts, Breyer, Kagan, Ginsburg and Sotomayor, as indicated by the FPC1 values in Fig 4. Though their policy position trajectories have been relatively stable, except for Chief Justice Roberts who exhibits a tendency to shift toward moderate, their views may still vary both over time and over different issue areas.

**Fig 10:**
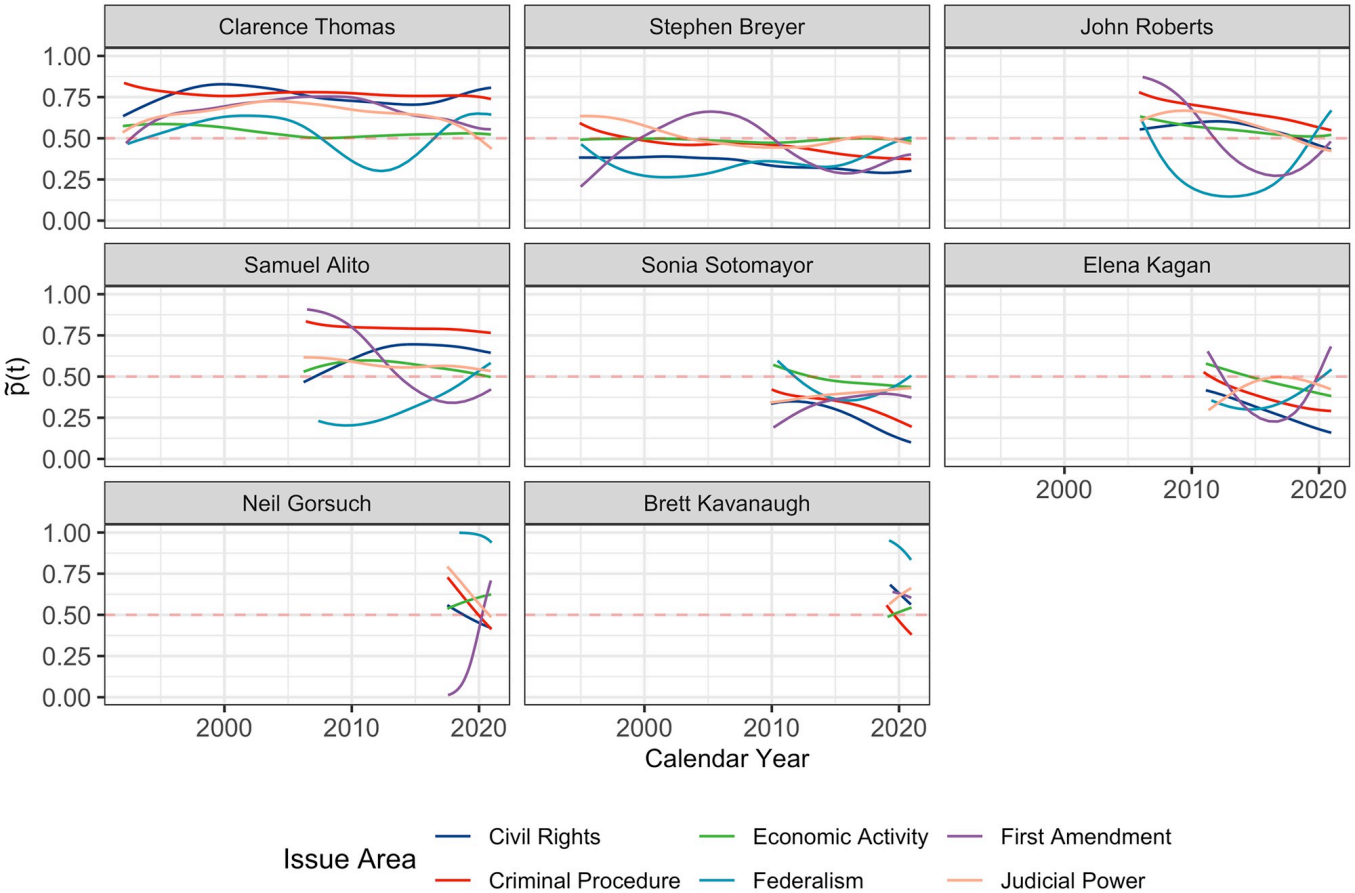
The inferred latent policy position processes $\tilde{p}\left(t\right)$ in the probability scale. These processes are obtained per Eqs (7) and (8) for the currently serving justices by issue areas, using only votes concerning selected issue areas: criminal procedure, civil rights, First Amendment, economic activity judicial power and federalism, respectively.

Specifically, cases before the court were labeled according to the following case categories: Criminal procedure, civil rights, First Amendment, due process, privacy, attorneys’ or governmental officials’ fees or compensation, unions, economic activity, judicial power, federalism, interstate relation, federal taxation, miscellaneous, and private law. The numbers of cases and relative frequencies for each issue area in the aggregate were: Criminal procedure (27,781, 11%), civil rights (23,834, 9.42%), First Amendment (7,175, 2.84%), due process (9,842, 3.89%), privacy (1,220, 0.482%), attorneys’ or governmental officials’ fees or compensation (2,967, 1.17%), unions (4,705, 1.86%), economic activity (73,684, 29.1%), judicial power (48,239, 19.1%), federalism (7,899, 3.12%), interstate relations (2,383, 0.942%), federal taxation (13,036, 5.15%), miscellaneous (994, 0.393%), and private law (28,248, 11.2%). These labels make it possible to conduct a more detailed analysis. The reconstructed latent policy position processes for selected issue areas are shown in Fig 10.

Rather than focusing on the between-justice difference in terms of overall policy position trajectories, the justice-area-specific ideology trajectories, obtained by applying the FPCA method to issue-specific votes, enable the study of within-justice differences, which is clearly present. The data suggests that there exists substantial variability even for the same justice across different issues. Votes for civil right, criminal procedure and judicial power cases and their underlying latent dispositions are found to be closely aligned with a justice’s overall ideological disposition. Furthermore, the justices are seen to be relatively more concordant regarding economic activity cases, where all nine justices are closer to the moderate center. The cases with the most divergent dispositions and discordant votes are those involving federalism and first amendment. Gorsuch and Kavanaugh have extremely conservative voting patterns on federalism cases, while the rest of the Court is mostly liberal, including other Republican justices. Dispositions regarding first amendment cases have shown some dynamics over calendar time, for example Roberts and Alito have drifted towards a higher percentage of liberal decisions in recent years, and also Gorsuch has been quite liberal in his votes. Area-specific analyses thus can provide further details and insights about the ideology of the justices.

Of additional interest is what would the ideology makeup of the Supreme Court be in the next few years. To this end, our methodology provides prediction of future ideology process based on the FPCA approach in the Section Functional principal component analysis (FPCA). This device has been recently also used for the prediction of COVID-19 case trajectories [37]. For more information, see S1 Appendix.

## Discussion

The ideology dynamics of the Supreme Court justices is of great interest especially under current circumstances when inequality and injustice in race, health, education and in other social aspects are at the frontline of societal discourse and there are substantial changes in the make-up of the Supreme Court. We have developed a functional data approach to analyze the latent policy position process using observable voting behaviors and demonstrated that this yields substantial insights in the time dynamics of policy preferences of the justices. Our approach is based on Functional Data Analysis (FDA), a nonparametric statistical methodology that is increasingly popular for the analysis of longitudinal studies or panel data (time- series cross-sectional (TSCS) data) and provides a suite of statistical models for analyzing dynamic behavior of stochastic processes, of which the ideology process is an instantiation.

The FDA approach makes it possible to compress high-dimensional trajectory data into a few concise variables, the functional principal components (FPCs), providing dimension reduction. The FPCs are directly interpretable in conjunction with the eigenfunctions, can be used for visualization and also may serve as a tool for further statistical analysis. For instance, judicial behavior and ideology might be an important explanatory factor for other political phenomena and for the prediction of Supreme Court votes. Our approach complements existing work related to judicial ideology and voting behavior.

The proposed approach has the advantages that it is truly dynamic as it recovers the entire trajectory of the latent policy position process for each justice, with weak requirements on the available data, allowing for discretely observed, noise contaminated data and provides predictions for individual justice’s ideological trajectories. Fast and timely updates of trajectories are readily available whenever votes on new cases are recorded and the approach facilitates the discovery of patterns of change over time. It does not require any a priori restrictive model assumptions or specification of presumed patterns. It turns out that it is sufficient to quantify an individual justice’s trajectory by just specifying two FPCs, where the first FPC corresponds to a mostly static and the second to a dynamic change component.

The proposed methodology provides a useful representation that incorporates substantial dimension reduction ofr the otherwise unwieldy observed longitudinal patterns. Implementation is straightforward through the established R package fdapace [36]. This package provides useful tools for the political science community to study repeatedly observed time-dynamic phenomena.

## Supporting information

S1 Appendix Prediction of future ideology processes for the justices in the current court. (PDF)
