# Supplementary material for: The dynamics of ideology drift among U.S. Supreme Court justices: A functional data analysis
Source: PLoS One. 2022 Jul 8;17(7):e0269598. doi: 10.1371/journal.pone.0269598 (PMC9269944; doi:10.1371/journal.pone.0269598)
Supplement: S1 Appendix — (PDF) [file pone.0269598.s001.pdf]

The dynamics of ideology drift among U.S. Supreme Court Justices: A functional data analysis

Supplementary Material

Xiner Zhou, Hans-Georg Müller

With the confirmation of Justice Amy Coney Barrett, there is a possible prospect of a shift given the new conservative 6-3 majority. After examining the past, can one utilize the historical data to predict what the ideological makeup of the Court would be like in the next 5 years? Our method has the ability to predict future ideology process for those Justices that have served less than 35 years and are considered as partially observed functional data, as detailed in the Method Section.

To quantify our model's ability to forecast future trajectory based on observed past trajectory, we conduct leave-one-out cross validation (LOOCV) to assess how well the model will generalize to out-of-sample data. Since our goal is to forecast the next 5 years, we select those judges with at least 5 years observations which results in 95 judges as our sample. One round of the LOOCV procedure involves partitioning the sample into a training set consisting of 94 judges and a testing set consisting the remaining 1 judge, and building the model using the training set only, and validating the prediction accuracy on the last 5 years of the testing judge. Lastly, an overall measure of the model's predictive

performance is calculated by averaging prediction accuracy over all rounds. To evaluate the prediction accuracy, we discretize both the smoothed trajectory of conservative votes $\hat{p}_i(t)$ and the model's predicted trajectory $\tilde{p}_i(t)$ at monthly grid. We use the mean squared prediction error (MSPE) between the discretized smoothed proportion of conservative votes and the discretized model predicted ideology in probability scale, as the measure of prediction accuracy. For ith round with ith judge as testing data, the MSPE is calculated as

$$\text{MSPE}_i = \frac{1}{u_i - l_i + 1} \sum_{j=l_i}^{u_i} [\hat{p}_i(t_{ij}) - \tilde{p}_i(t_{ij})]^2 \tag{1}$$

where l_i and u_i are the beginning index and ending index of the ith judge's last 5 years observations, respectively. Then the overall MSPE averaged over all cross-validations is 0.0009.

To better assess the validity of the model predictive performance, Fig **??** presents a selected 6 rounds of LOOCV results when the current judges with more than 5 years observation are used as the testing data. It shows that the forecast trajectories are tracking the target trajectory closely.

We then apply the model trained on all observed data to the current judges and predict their next 5 years likely ideology trajectories. According to our model's prediction, as shown in Fig **??**, it suggests the following possibilities. The liberal camp – Justices Sotomayor, Breyer and Kagan – will likely to stay where they are, with around 60%-70% of liberal decisions. The solid conservative camp – Justices Thomas and Alito – will also likely to stay where they are, with around 60%-70% of conservative decisions. Justices Gorsuch and Kavanaugh are projected to be moderately conservative. Chief Justice

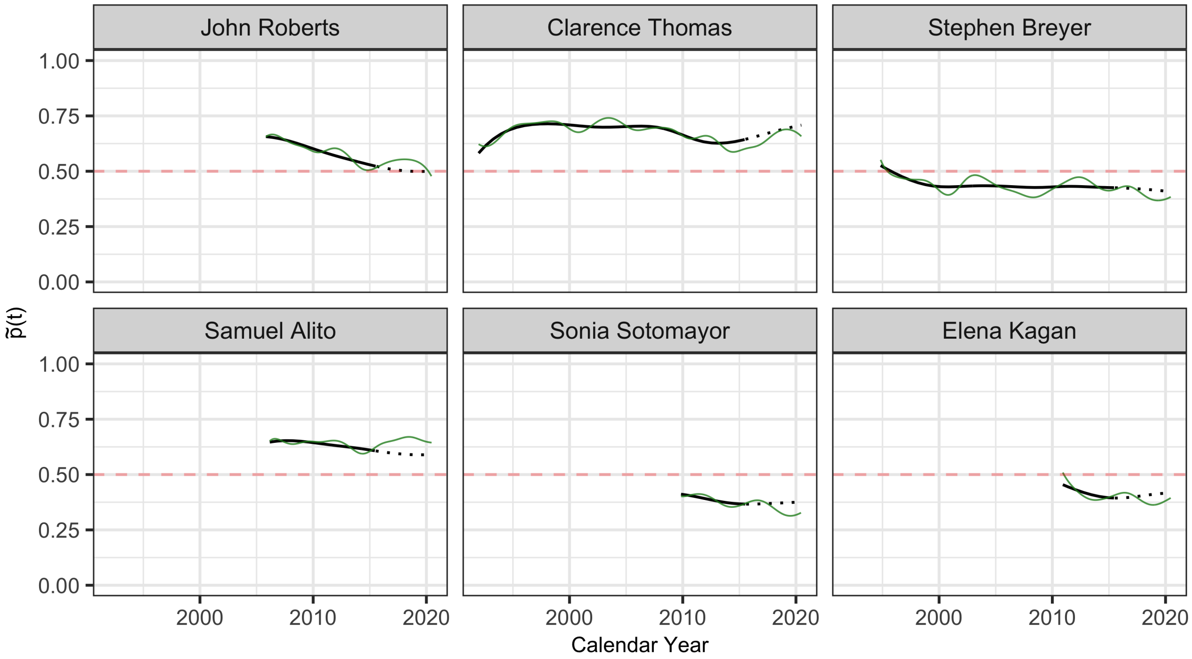

Figure 1: **LOOCV results when the current judges with more than 5 years observation are used as the testing data, respectively.** The solid green line represents the smoothed proportion of conservative votes $\hat{p}_i(t)$ against which accuracy is measured. The solid black lines represents estimated ideology trajectories corresponding to time periods during which there are observed voting data, while the dotted black lines represents prediction of future ideology trajectories that have yet not realized and are predicted based on past trajectory.

Roberts is projected to position almost at the center of the ideology space, with just a slight conservative inclination. The data-driven prediction should be viewed as suggestion and with caution as the models may not capture case contents and legal changes. Another wild card is the voting behavior of Justice Barrett, although early indicators put her into the same cluster as Justices Alito and Thomas. It also suggests the following ideology position to hold for the near future, from the most conservative to the most liberal (except Justice Barrett): Clarence Thomas, Samuel Alito, Neil Gorush, Brett Kavanaugh, John Roberts, Elena Kagan, Stephen Breyer, and Sonia Sotomayor. With Justice Stephen Breyer's decision to retire at the end of the current term (October term 2021), it gives

Democrats opportunity to replace the liberal justice and maintain the current 6-3 conservative majority in the court and also opens up possibilities of ideological shift of the court.

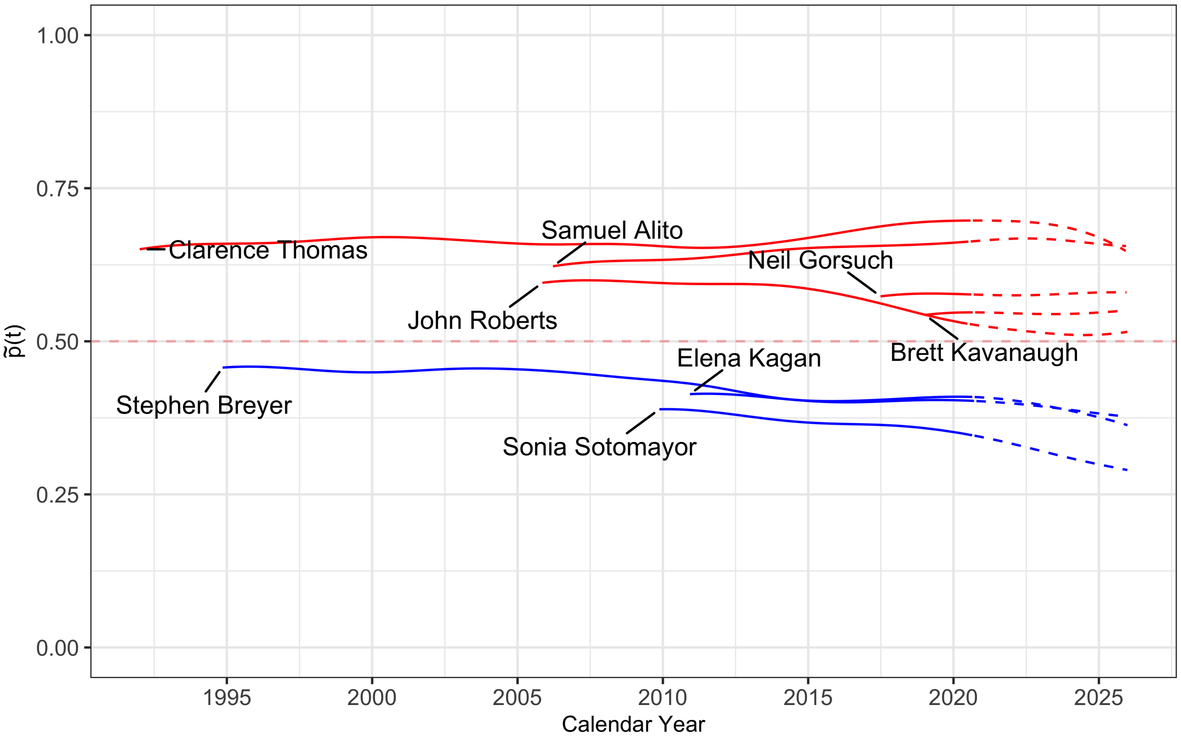

Figure 2: **Predicted ideology trajectories in the next 5 years (2021-2015) for the current Court (except Justice Barrett).** Color coded by the nominating president's party (blue for Democratic, red for Republican). The solid lines represents estimated ideology trajectories corresponding to time periods during which there are observed voting data, while the dashed lines represents prediction of future ideology trajectories that have yet not realized and based on past voting data.
